# Donor selection for adoptive immunotherapy with NK cells in AML patients: Comparison between analysis of lytic NK cell clones and phenotypical identification of alloreactive NK cell repertoire

**DOI:** 10.3389/fimmu.2023.1111419

**Published:** 2023-02-14

**Authors:** Raffaella Meazza, Loredana Ruggeri, Fabio Guolo, Paola Minetto, Paolo Canevali, Fabrizio Loiacono, Sara Ciardelli, Alessandra Bo, Silvia Luchetti, Alberto Serio, Letizia Zannoni, Christelle Retière, Natalia Colomar-Carando, Sarah Parisi, Antonio Curti, Roberto M. Lemoli, Daniela Pende

**Affiliations:** ^1^ Unità Operativa UO Immunologia, IRCCS Ospedale Policlinico San Martino, Genova, Italy; ^2^ Divisione di Ematologia e Immunologia Clinica, Dipartimento di Medicina, Ospedale Santa Maria della Misericordia, Università di Perugia, Perugia, Italy; ^3^ Clinica di Ematologia, Dipartimento di Medicina Interna (DiMI), Università degli studi di Genova, Genova, Italy; ^4^ Dipartimento di Ematologia e Oncologia, IRCCS Ospedale Policlinico San Martino, Genova, Italy; ^5^ Laboratorio Centro Cellule Staminali e Terapie Cellulari, IRCCS Ospedale Policlinico San Martino, Genova, Italy; ^6^ Dipartimento di Medicina Specialistica, Diagnostica e Sperimentale, Università di Bologna, Bologna, Italy; ^7^ Université de Nantes, Etablissement Français du Sang (EFS), Institut National de la Santé et de la Recherche Médicale (INSERM), Centre National de la Recherche Scientifique (CNRS), Centre de Recherche en Cancé rologie et Immunologie Intégrée Nantes Angers (CRCI2NA), Nantes, France; ^8^ IRCCS Azienda Ospedaliero-Universitaria di Bologna, Istituto di Ematologia “Seràgnoli”, Bologna, Italy

**Keywords:** natural killer cells (NK cells), donor selection, NK alloreactivity, killer immunoglobulin-like receptors (KIR), human leucocyte antigen (HLA), adoptive immunotherapy, acute myeloid leukemia (AML)

## Abstract

Natural killer (NK) cell-based adoptive immunotherapy in leukemia patients is an emerging field of interest based on clinical evidence of efficacy and safety. Elderly acute myeloid leukemia (AML) patients have been successfully treated with NK cells from HLA-haploidentical donors, especially when high amounts of alloreactive NK cells were infused. The aim of this study was comparing two approaches to define the size of alloreactive NK cells in haploidentical donors for AML patients recruited in two clinical trials with the acronym “NK-AML” (NCT03955848), and “MRD-NK”. The standard methodology was based on the frequency of NK cell clones capable of lysing the related patient-derived cells. The alternative approach consisted of the phenotypic identification of freshly derived NK cells expressing, as inhibitory receptors, only the inhibitory KIR(s) specific for the mismatched KIR-Ligand(s) (HLA-C1, HLA-C2, HLA-Bw4). However, in KIR2DS2^+^ donors and HLA-C1^+^ patients, the unavailability of reagents staining only the inhibitory counterpart (KIR2DL2/L3) may lead to an underestimated identification of the alloreactive NK cell subset. Conversely, in the case of HLA-C1 mismatch, the alloreactive NK cell subset could be overestimated due to the ability of KIR2DL2/L3 to recognize with low-affinity also HLA-C2. Especially in this context, the additional exclusion of LIR1-expressing cells might be relevant to refine the size of the alloreactive NK cell subset. We could also associate degranulation assays, using as effector cells IL-2 activated donor peripheral blood mononuclear cells (PBMC) or NK cells upon co-culture with the related patient target cells. The donor alloreactive NK cell subset always displayed the highest functional activity, confirming its identification accuracy by flow cytometry. Despite the phenotypic limitations and considering the proposed corrective actions, a good correlation was shown by the comparison of the two investigated approaches. In addition, the characterization of receptor expression on a fraction of NK cell clones revealed expected but also few unexpected patterns. Thus, in most instances, the quantification of phenotypically defined alloreactive NK cells from PBMC can provide data similar to the analysis of lytic clones, with several advantages, such as a shorter time to achieve the results and, perhaps, higher reproducibility/feasibility in many laboratories.

## 1 Introduction

Natural Killer (NK) cells, classified as CD3^–^CD56^+^ cells, are cytotoxic components of innate immunity that provide the first line of defense against neoplastic and viral infected cells (1). The delicate balance between activating and inhibitory signals, transmitted by an array of germline-encoded surface receptors upon engagement with specific ligands on neighboring cells, regulates the NK cell function (2). When these interactions result in activation, NK cells release the lytic granules containing perforin and granzymes towards target cells. Important checkpoint receptors are HLA-specific. During NK cell differentiation, NK cells become fully functional only when they express at least one inhibitory receptor capable of recognizing self-HLA (3, 4). This education process allows maintaining tolerance toward healthy autologous cells and sensing modification/loss of HLA class I expression on potential target cells (“missing self-recognition”) (5). Since the early stages, NK cells express CD94/NKG2A heterodimer, which recognizes the non-classical HLA-E, characterized by limited polymorphism (6). Then, they acquire the expression of KIR, a family of transmembrane proteins including both inhibitory (iKIR) and activating receptors (aKIR) (3, 7). KIR are composed of 2 or 3 Ig-like extracellular domains, a transmembrane region, and a long cytoplasmic tail containing ITIMs in iKIR (i.e., KIR2DL, KIR3DL) or a short cytoplasmic tail in aKIR (i.e., KIR2DS, KIR3DS). Certain iKIR recognize epitopes shared by distinct groups of HLA-A, -B, or -C molecules (termed KIR-ligands, KIR-L). In detail, KIR2DL1 is specific for HLA-C^Lys80^ allotypes carrying C2 epitope (i.e., HLA-C2), KIR2DL2/L3 recognize HLA-C^Asn80^ allotypes carrying C1 epitope (i.e., HLA-C1) but also with low-affinity HLA-C2, and KIR3DL1 is specific for HLA-B and HLA-A molecules sharing the Bw4 public epitope (i.e., HLA-Bw4). The best characterized aKIR is KIR2DS1, sharing the HLA-C2 specificity with KIR2DL1 (8, 9).

*KIR* genes are highly polymorphic and inherited as haplotypes, comprising centromeric (Cen) and telomeric (Tel) regions (10–12). Two major groups of *KIR* haplotypes have been defined, termed *A* and *B* (13, 14). *KIR A* haplotypes, composed by Cen-A/Tel-A, have a fixed and limited number of genes, and *KIR2DS4* as the only gene encoding aKIR, which is often represented by truncated/non-functional receptor in Caucasians (11, 15). Differently, *KIR B* haplotypes are characterized by large gene content diversity and by various genes encoding aKIR (8, 16).

The wide variety of KIR repertoires expressed by NK cells of different individuals is primarily determined by genetic factors due to the high polymorphism of *KIR* and *HLA* genotypes (8), and by environmental stimuli such as CMV exposure (17). At the single-cell level, KIR expression pattern results from stochastic events but is highly influenced by self-HLA class I molecules, following the rules of education. This kind of repertoire can produce alloreactive NK cells, characterized by the unique expression of iKIR(s) specific for autologous KIR-L(s), absent, however, in the allogeneic target cells, through “missing self-recognition” (3). In allogeneic hematopoietic stem cell transplantation (HSCT), donor NK cells can be alloreactive according to KIR/KIR-L mismatch in graft-versus-host (GvH) direction (18, 19). This model of NK alloreactivity is highly reliable, although alternative models have also been proposed (reviewed in (20). This analysis requires considering the HLA incompatibility between donor and recipient, the donor KIR repertoire, and KIR education (18, 21–23). Three types of NK alloreactivity can be identified on the basis of donor self-specific iKIR and the corresponding missing cognate HLA ligand in the recipient: Allo C1 (mediated by KIR2DL2/L3 and mismatched HLA-C1), Allo C2 (KIR2DL1 and mismatched HLA-C2), and Allo Bw4 (KIR3DL1 and mismatched HLA-Bw4) (23). Allo C1 is always possible because all donors have *KIR2DL2* and/or *KIR2DL3*, which are alleles of the same locus (11, 24). *KIR2DL1* is absent in ~3-5% of individuals, which cannot be Allo C2 donors (12). Allo Bw4 is impossible in donors characterized by two Tel-B regions (missing *KIR3DL1*, ~6% of the European population) or by the exclusive presence of *KIR3DL1* alleles (e.g. KIR3DL1*004) coding for molecules with Leu86 (instead of Ser86), which are retained in the cytoplasm (~12% of the population) (25, 26).

NK alloreactivity has already been employed in clinics, mainly to treat leukemia patients (22, 27, 28). T-cell-depleted HLA-haploidentical stem cell transplantation (haplo-HSCT) proved that donor NK alloreactivity was associated with better leukemia-free survival in acute myeloid leukemia (AML) patients without causing GvH disease (18, 21, 22, 29). Likewise, adoptive immunotherapy of AML patients using NK cells from haploidentical KIR-L-mismatched donors showed promising results (30, 31). Notably, the anti-leukemia effect correlated with the dose of infused alloreactive NK cells (“functional NK cell dose”) (32–34), whose predictive value for clinical outcome was confirmed in long-term analysis (35).

In this study, we evaluated NK alloreactivity of potential haploidentical donors for NK cell-based adoptive immunotherapy in a population of high-risk AML patients, recruited in two different clinical trials. We compared the frequencies of donor alloreactive NK cells analyzed by i) the study of the NK cell clones capable of lysing the related patient-derived cells (i.e., the refer methodology in the previous experience) and ii) the phenotypic identification of peripheral blood NK cells by the use of appropriate anti-KIR and anti-NKG2A monoclonal antibodies (mAb) combinations. The two approaches generally showed a good correlation with some interesting exceptions. These data support the use of the phenotypic approach to quantify the functional dose of NK cells, overcoming the various limitations related to the study of NK cell clones and cytotoxicity.

## 2 Material and methods

### 2.1 Patients and donors

Peripheral blood (PB) samples were obtained from AML patients (*n*=17) and their potential haploidentical donors (*n*=24) at IRCCS Ospedale Policlinico San Martino (Genoa, Italy) and IRCCS Azienda Ospedaliero-Universitaria S.Orsola-Malpighi (Bologna, Italy). All patients and donors provided written informed consent prior to participating. The present study was conducted in accordance with the Declaration of Helsinki. The patients were enrolled in two clinical trials: “NK-AML”, registered at ClinicalTrial.gov website (NCT03955848), and approved by the Local Ethics Committees CER-Liguria (Prot. n. 405REG2017) and CE-AVEC (Prot. n. 35/2017/O/Sper); “MRD-NK”, approved by CE-AVEC (Prot. n. 214/2018/Sper/AOUBo).

### 2.2 Description of NK therapy protocols: NK-AML and MRD-NK

The NK-AML multicenter clinical trial (NCT03955848) is based on the infusion of alloreactive NK cells as a consolidation strategy for adult leukemia patients. AML patients with high or intermediate risk *de-novo* or secondary disease, with age greater than 18 years, not eligible for HSCT due to medical contraindications or lack of donor, are eligible. AML of M3 FAB subtype are excluded from the study. The patients’ eligibility criteria require the presence of a haploidentical donor with alloreactive NK cells, and adequate renal, pulmonary and hepatic function. NK cell separation was performed using the CliniMACS system (Miltenyi Biotec) on total peripheral blood mononuclear cells obtained by leukapheresis from the selected donors (31, 32). After immunosuppressive chemotherapy, including fludarabine and cyclophosphamide as previously described (31), patients were infused intravenously with a single dose of cryopreserved NK cells (day 0) followed by subcutaneous administration of IL-2 (10 x 10^6^ IU/day, 3 times weekly; Novartis) for 2 weeks (6 doses total).

The MRD-NK clinical trial is based on the infusion of haploidentical KIR-L mismatched NK cells in adult AML patients, who are eligible for HSCT and achieved MRD-positive CR after conventional chemotherapy. Patients with active infections and/or abnormal renal, cardiac, pulmonary, and hepatic function and/or poor performance status are excluded. Immunosuppressive chemotherapy and IL-2 administration in this trial were superimposable to NK-AML.

### 2.3 Cell lines

The erythroleukemia K562 and the AML MOLM-14 cell lines were cultured in RPMI 1640 medium (Lonza) supplemented with 10% FBS (Euroclone), 2 mM L-glutamine (Lonza), and 100 U/mL penicillin-streptomycin (Lonza) in 5% CO2 incubator at 37°C. All cell lines were routinely screened for mycoplasma infection by PCR analysis and used within four passages in culture after thawing.

### 2.4 KIR-ligand and *KIR* genotype

HLA typing was performed in patients and relatives searching for haploidentical NK alloreactive donors, following the model of KIR/KIR-L mismatch in GvH direction. HLA-B and HLA-C alleles were analyzed using the KIR-ligand calculator program (http://www.ebi.ac.uk/ipd/kir/ligand.html ), to define HLA-C1, HLA-C2, and HLA-Bw4, implementing with other notions, as described in the Results section (paragraph 3.1). Potential NK alloreactive donors were identified by the presence of KIR-L(s) missing instead in the related patient. The presence of the iKIR implicated in the recognition of the missing ligand and the functional protein was confirmed by cytofluorimetric analysis (see below) and molecular genotyping. DNA was extracted from the blood of selected donors using a commercially available kit (Macherey-Nagel). *KIR* genotype was analyzed using Olerup SSP-PCR KIR genotyping KIT (CareDx) according to the manufacturer’s instructions.

### 2.5 PBMC isolation and PHA-blast derivation

PBMC from patients and haploidentical donors were isolated by Lympholyte-H (Cedarlane Laboratories) density-gradient centrifugation. To obtain PHA-blasts, PBMC were stimulated with the mitogen phytohemagglutinin-M (PHA-M, 1μg/mL; Sigma-Aldrich) and cultured in RPMI 1640 medium (Lonza) supplemented with 10% FBS (Euroclone), 2 mM L-glutamine (Lonza), 100 U/mL penicillin-streptomycin (Lonza) and Interleukin (IL)-2 (300IU/ml) (Proleukin, Clinigen Healthcare Ltd.).

### 2.6 NK cell purification, cloning, and cytotoxicity

Donor NK cell isolation was performed using peripheral whole blood donor samples by human NK isolation KIT (Miltenyi Biotec). Donor NK cells were plated under limiting-dilution conditions, activated with PHA, and cultured with IL-2 and irradiated feeder cells. Feeder cells were obtained by pooling buffy coats from 5 to 9 healthy donors. We used clones with frequencies ≤ 20 clones/plate. The cytotoxic capacity of the clones was assessed by standard ^51^Cr-release assay against K562 target cell line. NK clones that killed K562 were screened for alloreactivity at an effector-to-target ratio of 10:1 against patient KIR-L mismatched or autologous PHA lymphoblasts. Clones exhibiting more than 30% lysis against patient target cells were scored as alloreactive.

### 2.7 Cytofluorimetric analysis of KIR and NK alloreactive subsets

The surface phenotyping of NK cells (gating CD3^–^CD56^+^ cells) was performed on donor-derived PBMCs, mononuclear cells from leukapheresis, purified NK cells, or NK cell clones by multi-parametric flow cytometry. All mAbs used in this study are detailed in **Supplementary Table 1** and **Supplementary Table 2**. For all the stainings, 2 x 10^5^ cells were used in each sample. Indirect stainings were performed using ECM-41 (36, 37) and 1F12 (38) mAbs followed by anti-mouse IgM-FITC and anti-mouse IgG2b-PE, respectively, secondary reagents (Southern Biotech). To discriminate relevant iKIR and aKIR, we used EB6-PE/143211-FITC (anti-KIR2DL1/S1 and anti-KIR2DL1), GL183-PE/ECM-41-FITC (anti-KIR2DL2/L3/S2 and anti-KIR2DL3), GL183-APC/ECM-41-FITC/1F12-PE (anti-KIR2DL2/L3/S2 and anti-KIR2DL3 and anti-KIR2DL3/S2), Z27-APC/DX9-PE-Vio770 (anti-KIR3DL1/S1 and anti-KIR3DL1) combinations. For identifying the three different NK cell alloreactive subsets (Allo C1, Allo C2, and Allo Bw4), the appropriate mAb combinations used are described in **Supplementary Table 2**. All the incubations with antibodies were performed at 4°C for 30 minutes, thereafter the cells were washed in PBS (Lonza) with 2% FBS (Euroclone). Samples were acquired using Gallios (Beckman Coulter) or MACSQuant-analyzer (Miltenyi-Biotech) and data analyzed with FlowJo, Version 10.7 (BD Biosciences).

### 2.8 Degranulation assay

Donor PBMCs (or purified NK cells), cultured for 3-5 days in the presence of IL-2 (600IU/mL), were incubated at 37°C for 3 hours with or without target cells (patient PHA-blasts, MOLM-14, or K562) at E:T of 2:1 (2 x 10^5^ effector and 1 x 10^5^ target cells), and anti-CD107a-PE mAb. Golgi Stop (BD Biosciences) was added after the first hour of incubation. Thereafter, cells were collected, washed in PBS with 2% FBS and 2mM EDTA, and stained (30 minutes at 4° C) with anti-CD3-BV510, -CD56-BV421, -CD107a-PE, and the appropriate antibody combinations allowing the identification of the different NK cell subsets (**Supplementary Tables 1** and **3**). Samples were analyzed by Gallios flow-cytometer (Beckman Coulter). Data analysis was performed using FlowJo Version 10.7 (BD Biosciences); the gating strategy is shown in **Supplementary Figure 1**. Data referred as Δ CD107a represent the difference between the % of CD107a^+^ NK cells co-cultured with target cells and the % of CD107a^+^ NK cells cultured with medium alone.

### 2.9 Statistical analysis

Graphical representation and statistical analysis were performed with Prism software, Version 9.0.2 (GraphPad Software). Kruskal-Wallis followed by Dunn’s multiple comparison test was used to analyze experiments with more than two groups. Correlation analysis and correlation graphs were performed with IBM SPSS^®^ v22, with the Pearson’s R correlation coefficient. Not significant (ns); ****p< 0.0001; ***p< 0.001; **p< 0.01; and *p< 0.05. *n* is the number of samples used in the experiments. The means are shown, and bars indicate SEM.

## 3 Results

### 3.1 Selection of haploidentical donors by NK alloreactivity

We analyzed the haploidentical donors of 13 and 4 patients recruited in the NK-AML and MRD-NK clinical trials, respectively (**Table 1**). HLA typing and the KIR-ligand calculator results of AML patients and family members allowed identifying potential NK alloreactive donors, if a KIR-L mismatch in GvH direction was detected. Among the 17 patients, 11 had a single and 6 had multiple KIR-L mismatched donors (**Table 1**). As already described (23), we also considered other notions, mainly consisting in Bw4 alloreactivity peculiarities. We found in six individuals the presence of B*13:02, a Bw4 allele that, however, is not a ligand of KIR3DL1 (39). This observation led us questioning the NK alloreactivity of the two donors (D1 and D2) for patient #15, nevertheless, we decided to proceed to further analyses. In addition, we considered the HLA-A alleles with Bw4 epitope, which are recognized by KIR3DL1 (i.e., HLA-A*23, -*24, and -*32 (39, 40). The inclusion of HLA-A*32 as Bw4 in patient #12 led us to evaluate only C2 mismatch for both donors, not considering the double mismatch (Bw4 and C2). We also considered the C1 epitope of B*46:01 and B*73:01 (41). Only one donor (D of patient #7) has one of these alleles (B*73:01), without changing the KIR-L pattern due to the presence of HLA-C1.

**Table 1:** KIR-ligands of patients and their potential haploidentical donors.

Patient			Donor			
#	UPN	KIR-L	ID	Family member	KIR-L	Mismatched KIR-L^§^
1	NK-AML_005	C1/C2	D1	sister	Bw4, C1/C2	Bw4
			D2	son1	Bw4, C1/C2	Bw4
			D3	son2	Bw4, C1/C2	Bw4
2	NK-AML_008	Bw4, C1/C1	D	son	Bw4, C1/C2	C2
3	NK-AML_010	Bw4^, C2/C2	D	daughter	Bw4^, C1/C2	C1
4	NK-AML_015	Bw4, C1/C1	D	son	Bw4, C1/C2	C2
5	NK-AML_012	C1/C1	D	son	Bw4, C1/C1	Bw4
6	NK-AML_018	C1/C2	D	daughter	Bw4, C1/C2	Bw4
7	NK-AML_019	Bw4, C1/C1	D	son	Bw4, C1/C2	C2
8	NK-AML_020	Bw4^, C2/C2	D1	daughter1	C1/C2	C1
			D2	daughter2	C1/C2	C1
9	NK-AML_023	Bw4, C2/C2	D1	daughter1	Bw4, C1/C2	C1
			D2	daughter2	Bw4, C1/C2	C1
10	NK-AML_026	Bw4, C1	D	son	Bw4, C1/C2	C2
11	NK-AML_025	Bw4, C2/C2	D1	son	Bw4, C1/C2	C1
			D2	nephew	Bw4, C1/C2	C1
12	NK-AML_031	Bw4^#^, C1/C1	D1	daughter1	Bw4, C1/C2	C2
			D2	daughter2	Bw4, C1/C2	C2
13	NK-AML_032	Bw4, C2/C2	D	brother	Bw4, C1/C2	C1
14	MRD-NK_001	C1/C2	D	mother	Bw4, C1/C2	Bw4
15	MRD-NK_002	C2/C2	D1	daughter	Bw4^, C2/C2	Bw4^
			D2	son	Bw4^, C2/C2	Bw4^
16	MRD-NK_003	Bw4, C2/C2	D	sister	Bw4^, C1/C2	C1
17	MRD-NK_004	C1/C2	D	daughter	Bw4, C1/C2	Bw4

D indicates when a unique donor was available, while D1, D2, or D3 when alternative donors were tested.

**^§^** KIR-L present in the donor and missing in the patient.

Bw4^ indicates when the unique Bw4 epitope is represented by B*13:02, an allele that is not a ligand of KIR3DL1

Bw4^#^ indicates when the unique Bw4 epitope is represented by A*32:01, an allele that is a ligand of KIR3DL1

### 3.2 Analysis of donor KIR phenotype and genotype

**Figure 1:**
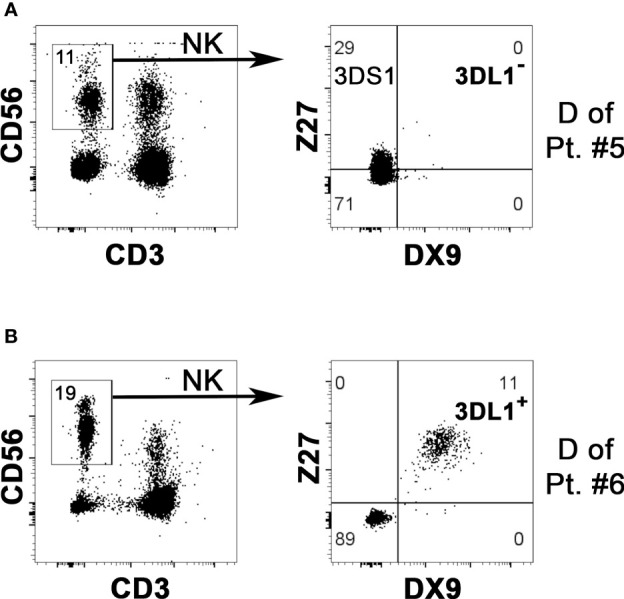
Evaluation of KIR3DL1 NK surface expression to confirm possible NK alloreactivity in case of HLA-Bw4 mismatch. By flow-cytometry, NK cells (gating on CD3^–^ CD56^+^ cells of PBMC, left panels) from two potential Allo Bw4 donors were analyzed using DX9 (anti-KIR3DL1) and Z27 (anti-KIR3DL1/S1) mAbs to detect the surface expression of KIR3DL1 (i.e., the relevant iKIR specific for HLA-Bw4) and/or KIR3DS1 (right panels). The “KIR” acronym has been omitted in the receptors identified within the quadrants. **(A)** NK cells from donor of patient #5 did not express KIR3DL1 (DX9^–^), while KIR3DS1 (Z27^+^/DX9^−^) was present. This donor couldn’t be considered as NK alloreactive. **(B)** a portion of NK cells from the donor of patient #6 expressed KIR3DL1 (Z27^+^/DX9^+^), confirming the NK alloreactivity.

**Figure 2:**
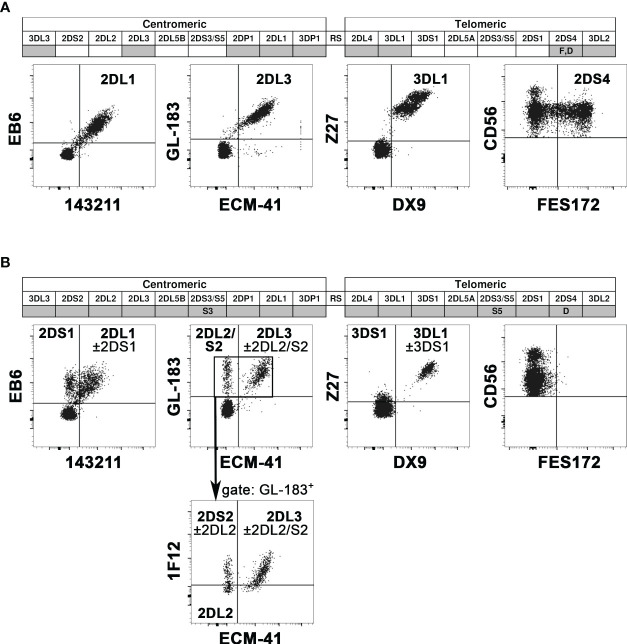
Representative analyses of *KIR* gene profiles and KIR phenotype. In the *KIR* gene profiles (top), the *KIR* presence or absence is indicated with grey or white boxes, respectively. *KIR2DS4* alleles coding for surface receptors are reported as F, while those coding for truncated receptors as D. *KIR2DS3* and *KIR2DS5* are indicated by S3 and S5, respectively. Cytofluorimetric analysis of PBMC (bottom) shows the surface expression of various iKIR and aKIRs on NK cells (gating on CD3^−^ CD56^+^ cells), using different mAb combinations (see mAb specificity in **Supplementary Table 1**). The “KIR” acronym has been omitted for both genes and molecules. Two representative donors were evaluated. **(A)** D1 of patient #12 was characterized by *KIR* A/A genotype and the expression of all analyzed iKIR, and only KIR2DS4 as aKIR. **(B)** D2 of patient #8 was characterized by *KIR* B/x genotype, and the expression of all detectable iKIR and aKIR (excepted KIR2DS4). Gating on GL183^+^ NK cells, 1F12/ECM-41 mAb combination also allowed the discrimination of KIR2DS2^+^, KIR2DL2^+^, and KIR2DL3^+^ NK cell subsets.

We evaluated the KIR phenotype of NK cells from all potential donors, while *KIR* genotype analysis was applied only to the donors selected for the NK cell infusion. First of all, KIR phenotype allows detecting on the NK cell surface the expression of the KIR specific for the KIR-L mismatch relevant for the alloreactivity. Particularly crucial is detecting KIR3DL1 for Bw4 mismatch because it can be missing in a substantial proportion of individuals. The cytofluorimetric analysis using Z27 (anti-KIR3DL1/S1) mAb in combination with DX9 (anti-KIR3DL1) mAb allows discriminating KIR3DL1 from KIR3DS1. NK cells from the D of patient #5 expressed KIR3DS1 and not KIR3DL1 (**Figure 1A**). The lack of KIR3DL1 in this donor led to the absence of the Allo Bw4 type of NK alloreactivity. Thus, we excluded this case from the cohort of patients for NK cell infusion, avoiding further analyses. Differently, in the other potential Allo Bw4 donors the presence of KIR3DL1 was confirmed. A representative donor with KIR3DL1^+^ KIR3DS1^−^ NK cells (D of patient #6) is shown in **Figure 1B**. In addition to this staining, other mAb combinations allow the detection of relevant iKIR and aKIR. We can distinguish KIR2DL1 from KIR2DS1 using 143211/EB6 mAb, KIR2DL3 from KIR2DL2/S2 using ECM-41/GL-183 mAb combinations, and we can detect KIR2DS4 with FES172 mAb. When analyzed, the KIR genotype always confirmed the hypothesis of the presence/absence of these mentioned KIRs. Two representative donors are shown in **Figure 2**. NK cells from D1 of patient #12 (**Figure 2A**) showed expression of the iKIR as KIR2DL1, KIR2DL3, and KIR3DL1, and only KIR2DS4 as aKIR, a pattern consistent with the identified *KIR* A/A genotype. Differently, on NK cells from D2 of patient #8 (**Figure 2B**), KIR2DL1, KIR2DL2, KIR2DL3, and KIR3DL1 as iKIR, in addition to KIR2DS1, KIR2DS2, and KIR3DS1 as aKIR, were detected. Using GL-183/ECM-41/1F12 mAb combination, we revealed the presence of KIR2DL2^+^ (GL-183^+^ECM-41^−^1F12^−^), KIR2DS2^+^ (GL-183^+^ECM-41^−^1F12^+^), and KIR2DL3^+^ (GL-183^+^ECM-41^+^1F12^+^) cell subsets. The KIR phenotype of D2 of patient #8 was consistent with the *KIR* B/x genotype, composed of Cen-A/Cen-B and Tel-A/Tel-B regions with KIR2DS4 delta, as confirmed by the complete *KIR* gene profile analysis.

By KIR analyses, all the evaluated donors except for one were NK alloreactive, and the iKIR and aKIR repertoire of selected donors has been identified.

### 3.3 Quantification of the NK alloreactive subset by flow cytometry

**Figure 3:**
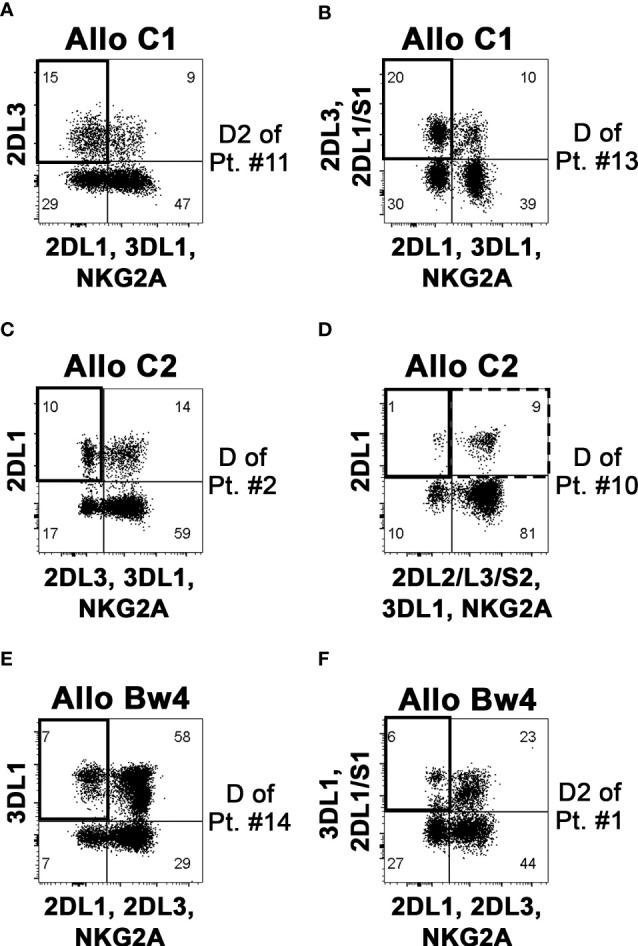
Identification and quantification of the alloreactive NK cell subset on donor PBMC. By flow-cytometry, NK cells (gating on CD3^–^ CD56^+^ cells of PBMC) were analyzed to define the size of the alloreactive subset (indicated as a square in upper left quadrants). Representative donors characterized to be Allo C1 **(A, B)**, Allo C2 **(C, D)**, and Allo Bw4 **(E, F)**, considering the KIR-L present in the donor and missing in the recipient and the presence of donor iKIR specific for the mismatched KIR-L, are shown. Three donors were without (**A, C, E**), and three donors with the relevant aKIR **(B, D, F)** as indicated in **Table 2**. The presence of KIR2DS2 can determine an underestimation of the alloreactive NK cell subset, as shown for the Allo C1 donor of patient #10 **(D)**; the square with a dashed line (upper right quadrant) includes the possible presence of alloreactive KIR2DL1^+^ KIR2DS2^+^ cells that cannot be properly quoted.

From the notion of the mismatched KIR-L (i.e., present in the donor and missing in the recipient), shown in **Table 1**, and with the identification in donor NK cells of the iKIR specific for the mismatched KIR-L, we could divide the patients into three groups based on the different donor NK alloreactivity: Allo C1, Allo C2, and Allo Bw4 (**Table 2**). There were no donors with a double kind of alloreactivity. As already stated, patient #5 was excluded from Allo Bw4 group, because the respective potential donor was KIR3DL1^−^. Analyzing the pattern of iKIR and aKIR on NK cells from all donors, it was possible to define the appropriate mAb combinations to detect the alloreactive subsets, expressing as inhibitory receptor only the iKIR specific for the KIR-L mismatch (**Supplementary Table 2**). The presence of aKIR can contribute to the alloreactive subset. This is particularly true for KIR2DS1 in HLA-C1^+^ donors and HLA-C2^+^ patients, which is considered “educated and useful”, so KIR2DS1 can be included in the alloreactive NK cell subset. In contrast, it is not possible to quote KIR2DS2 in Allo C2 and Allo Bw4 (particularly with HLA-C1^+^ patients), because there is no availability of mAb specific only for the inhibitory counterpart (KIR2DL2 and eventually KIR2DL3) and not reacting also with KIR2DS2. This phenotypic limitation leads to underestimating the alloreactive NK cell subset. Representative cases for the three types of alloreactivity are shown in **Figure 3**. Cytofluorimetric analyses with different mAb combinations allowed the detection of the alloreactive subsets in Allo C1 (**Figures 3A, B**), Allo C2 (**Figures 3C, D**), and Allo Bw4 (**Figures 3E, F**). NK cells from donors characterized by the absence (**Figures 3A, C, E**) or the presence of the relevant aKIR (**Figures 3B, D, F**) were evaluated. In the absence of relevant KIR2DS1 and KIR2DS2, the frequency of single-KIR2DL3^+^, single-KIR2DL1^+^, and single-KIR3DL1^+^ NK cells allowed revealing the size of Allo-C1, Allo-C2, and Allo-Bw4 subsets, respectively (**Figures 3A, C, E**). In the case of *KIR2DS1* ^+^ Allo C1 donors, we also used the EB6 (anti-KIR2DL1/S1) mAb together with GL183 (anti-KIR2DL2/L3/S2) mAb, conjugated with the same fluorochrome. This staining combined with 143211 mAb conjugated with a different fluorochrome allows to exclude the KIR2DL1 inhibitory receptor (**Figure 3B**). Similarly, in Allo Bw4 and *KIR2DS1* ^+^ donors, Z27 (anti-KIR3DL1/S1) mAb staining was associated to EB6 mAb (**Figure 3F**). Conversely, in Allo C2 and *KIR2DS2* ^+^ donors, KIR2DL1^+^ KIR2DS2^+^ couldn’t be quoted in the alloreactive subset together with single-KIR2DL1^+^ NK cells because no mAb is reacting exclusively with KIR2DL2 or KIR2DL2/L3. In these cases, the size of the alloreactive subset may be underestimated. In Allo Bw4, no *KIR2DS2* ^+^ donor and HLA-C1^+^ patient combination was present in our cohort. In a group of donors, we also evaluated the size of the alloreactive NK cell subset in mononuclear cells from leukapheresis and NK cells separated by CliniMACS procedure. The results were consistent with those obtained on PBMC (**Supplementary Figure 2**). In addition, we performed further staining with the anti-LIR1 mAb. This mAb was included to detect the possible contribution of this additional inhibitory receptor (42) (**Supplementary Figure 3**). **Table 2** shows the data summarizing the size of the phenotypically defined NK alloreactive subsets for all donors, eventually excluding LIR1^+^ cells.

**Table 2:** NK alloreactive subsets and relevant KIRs in the various donor/patient pairs.

ALLO	Patient	Donor ID	Relevant iKIR	aKIR (2DS1, 2DS2)^	% of NK alloreactive subset^§^	% of NK alloreactive clones
**C1**	3	D	2DL2	2DS2	48 (13)	3
	8	D1	2DL2/L3	**2DS1**, 2DS2	23 (14)	13.6
		**D2**	2DL2/L3	**2DS1**, 2DS2	15 (7)	6.3
	9	**D1**	2DL3	**2DS1**, 2DS2	22 (15)	3
		D2	2DL2/L3	**2DS1**, 2DS2	16 (8)	1
	11	D1	2DL2/L3	**2DS1**, 2DS2	8 (4)	4
		**D2**	2DL3	−	15 (11)	6
	13	D	2DL3	**2DS1**	20 (1)	2.6
	16	D	2DL2/L3	2DS2	12 (5)	6
**C2**	2	D	2DL1	2DS1	10 (5)	10
	4	D	2DL1	**2DS2**	2 (1)	3.5
	7	D	2DL1	2DS1, **2DS2**	2 (1)	2
	10	D	2DL1	**2DS2**	1 (1)	18
	12	**D1**	2DL1	−	9 (6)	7
		D2	2DL1	−	5 (4)	2
**Bw4**	1	**D1**	3DL1	−	7 (5)	2.5
		D2	3DL1	**2DS1**	6 (2.5)	1.5
		D3	3DL1	**2DS1**	3 (1)	1.5
	6	D	3DL1	−	2 (1)	3
	14	D	3DL1	−	7 (6)	10
	15	D1	3DL1	2DS2	2 (1)	0
		D2	3DL1	2DS2	2 (1)	0
	17	D	3DL1	−	3 (2)	2.5

Related to each patient, D indicates a single donor, while D1, D2, or D3 multiple donors, with the chosen one written in bold.

**^**KIR2DS1 is written in bold when it can contribute to the alloreactive subset (i.e., when HLA-C1^+^ donor and HLA-C2^+^ patient); KIR2DS2 is written in bold when it can contribute to the alloreactive subset but cannot be quoted by phenotypic analysis (i.e., in Allo C2 or Allo Bw4 when HLA-C1^+^ patient).

**^§^** Percentages of phenotypically defined alloreactive NK cells (gating CD3^−^ CD56^+^ cells in PBMC) are reported. Numbers within brackets refer to alloreactive subsets after the further subtraction of LIR1^+^ cells.

### 3.4 Analysis of the functional activity of alloreactive versus non-alloreactive NK cell subset

**Figure 4:**
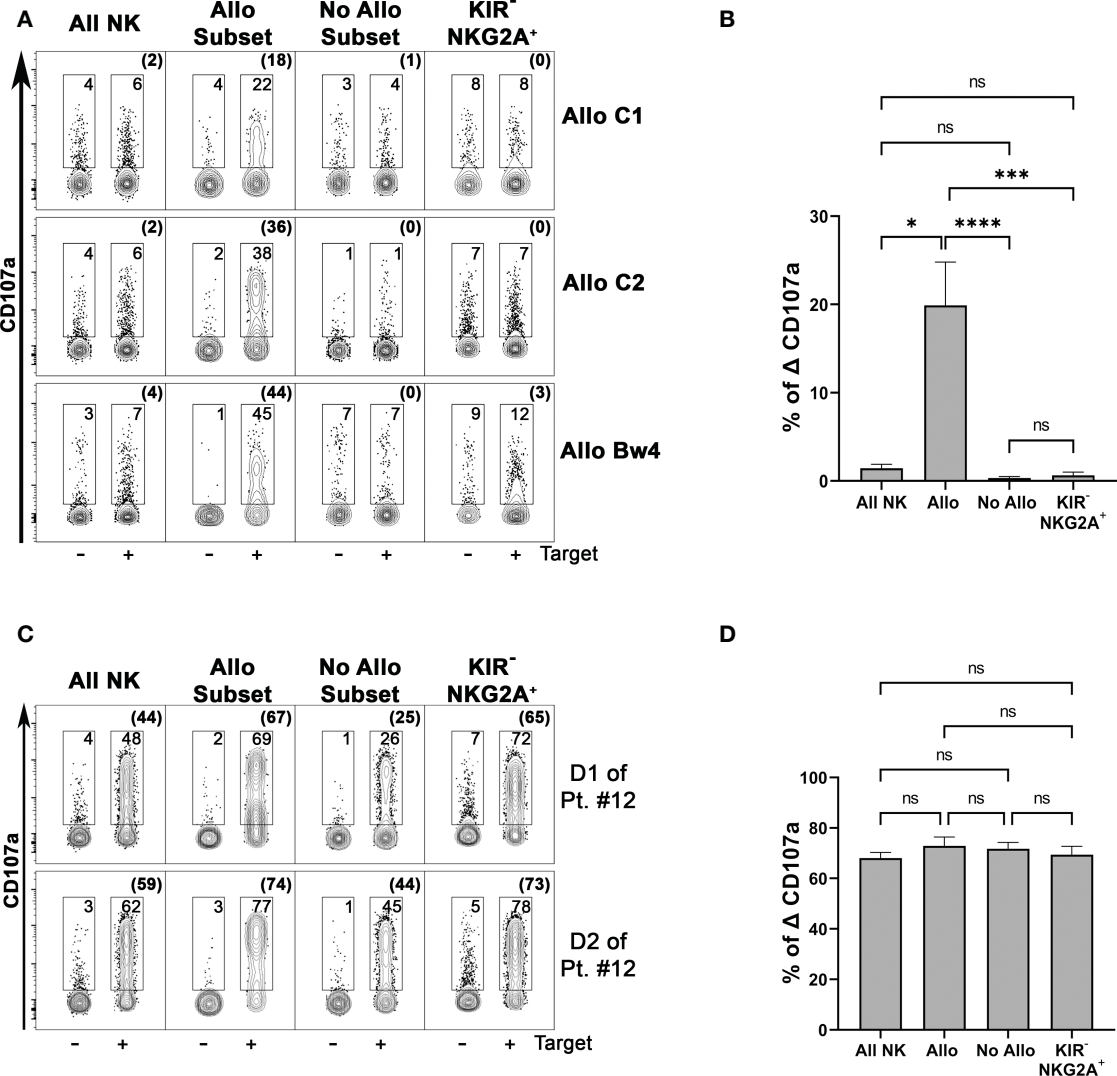
The phenotypically identified donor alloreactive NK cell subsets display the highest degranulation capability against the related patient target cells. **(A)** Degranulation activity of all NK (CD3^–^ CD56^+^ cells) and different NK cell subsets from three donors representative for each type of alloreactivity (D of patient #16, D1 of patient#12, D of patient#14) evaluated upon incubation in the absence or in the presence of PHA-blasts derived from the related patients (Target). Allo Subset consists of NK cells expressing only the iKIR specific for the KIR-L that is missing in target cells, No Allo Subset represents NK cells expressing iKIRs specific for KIR-L on target cells, and KIR^−^ NKG2A^+^ represents NK cells expressing only NKG2A and no KIRs. E:T ratio 2:1. Numbers indicate the percentage of surface CD107a^+^ cells, and the percentages of Δ CD107 are indicated in brackets. The samples of each donor were concatenated (data aggregated into one file) and analyzed using Flowjo. **(B)** Cumulative data of specific NK cell degranulation activity (Δ CD107a) from different donors after stimulation with patient-derived PHA-blasts. Different NK cell subsets were considered. E:T ratio 2:1. *n*=9 (Allo C1 = 2, Allo C2 = 2, Allo Bw4 = 5). Not significant (ns), *p< 0.05, ***p< 0.001, and ****p< 0.0001 (Kruskal-Wallis followed by Dunn’s multiple comparison test). Mean + SEM are reported. **(C)** Degranulation activity of different NK cell subsets from two representative Allo C2 donors was tested upon incubation in the absence or in the presence of the MOLM-14 target cell line. **(D)** Specific degranulation activity (Δ CD107a) of NK cells from different donors after stimulation with K562 cell line. *n*=10 (Allo C1 = 6, Allo Bw4 = 4). E:T ratio 2:1.

We tested NK cell alloreactivity in functional degranulation assays against patient-derived PHA-blasts, using as effector cells PBMC (or NK cells) from the related donors after short-term activation with IL-2. Specific CD107a surface expression on different NK cell subsets could be evaluated upon co-culture with the target cells. Representative cases for the three types of NK alloreactivity and cumulative data are shown in **Figures 4A, B**. Importantly, the alloreactive NK cell subset showed the highest CD107a surface expression, significantly different from those detected on other non-alloreactive subsets, expressing either iKIR recognizing patient KIR-L or NKG2A. The evaluation of the total NK cell population revealed only a very low degranulation. In Allo C2 donors, we also tested as target cells the AML cell line MOLM-14 characterized by the presence of HLA-C1 and HLA-Bw4 as KIR-L. Although this cell line efficiently stimulated the degranulation of the whole NK cell population, also in this case the NK alloreactive subset displayed the highest degranulation ability (**Figure 4C**). Moreover, the degranulation assay against the HLA class I negative K562 proved that both alloreactive and non-alloreactive subsets were equally functional (**Figure 4D**).

These functional data support our approach of phenotypic identification of the NK alloreactive subset endowed with higher activity versus patient-derived cells.

### 3.5 Comparison of the two approaches to define the frequency of alloreactive NK cells

**Figure 5:**
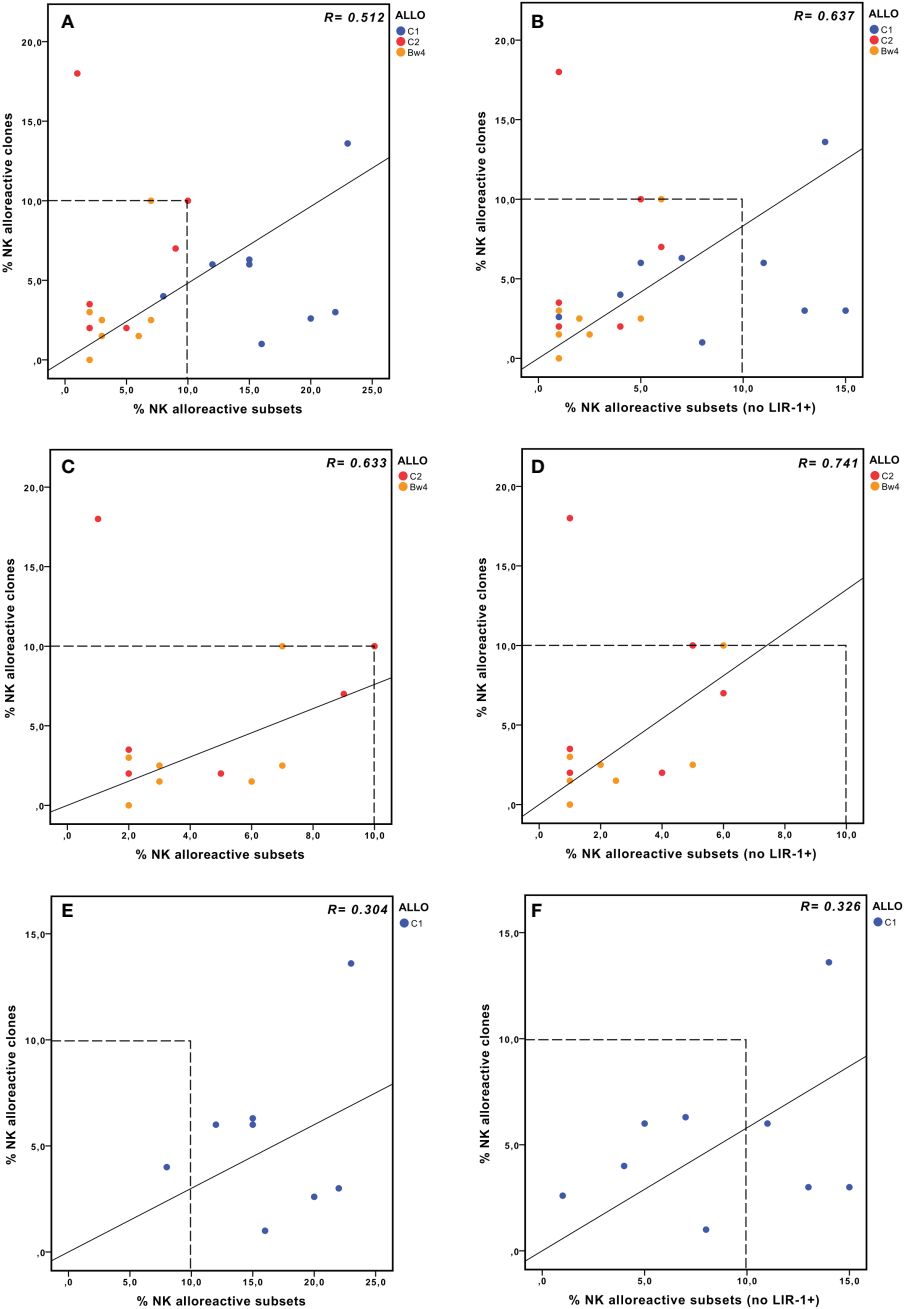
NK alloreactivity data from cytofluorimetric analysis of PBMC and lytic NK cell clones. Correlation analysis between the values obtained by the two different approaches, considering either the whole cohort **(A, B)**, Allo Bw4, and Allo C2 only **(C, D)**, or Allo C1 only **(E, F)**. The size of the alloreactive NK cell subset phenotypically defined without **(A, C, E)** or with the further exclusion of LIR1^+^ cells **(B, D, F)**. In panels (**A, E**) the % NK alloreactive subset of D of patient #3 (Allo C1, 48% without the exclusion of LIR1^+^ cells) is not shown because its value is out of the range of the axis, but it was considered in the correlation analysis.

**Figure 6:**
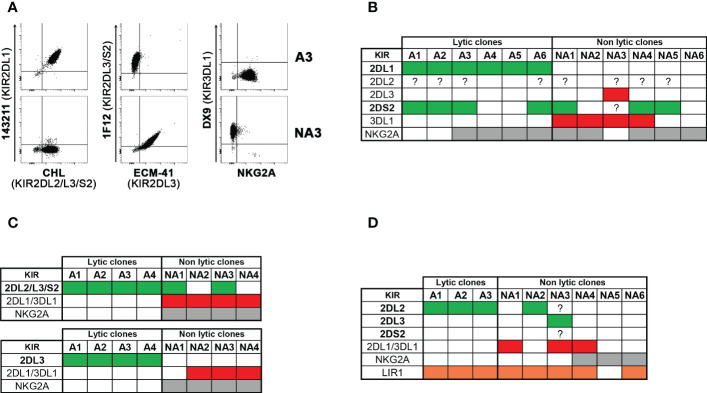
Cytofluorimetric analysis of lytic versus non lytic NK cell clones. NK cell clones derived from four donors, selected to be lytic (alloreactive, A) and non-lytic (non-alloreactive, NA) against the related patient PHA-blasts were analyzed, by flow cytometry, for the expression of relevant KIRs, NKG2A, and in one case LIR1. Considering the type of alloreactivity, the presence of the permissive iKIR(s) and relevant aKIR are written in bold and indicated as green, the blocking iKIR(s) as red, NKG2A as grey, and LIR1 as orange boxes, while their absence as white boxes; a question mark indicates uncertainty due to the lack of mono-specificity of certain mAbs (i.e., GL-183, CH-L and 1F12). Representative stainings **(A)** and cumulative data **(B)** of clones derived from D of patient #10 (Allo C2). **(C)** summary of clones from D1 (up) and D2 (bottom) of patient #11 (Allo C1). **(D)** summary of clones from D of patient #16 (Allo C1).

We compared the size of the phenotypically defined alloreactive NK cell subset with the frequency of NK cell clones capable of lysing patient-derived PHA-blasts in cytotoxicity assays (i.e., lytic clones), considered the reference methodology in previous studies (31, 32) (**Table 2**). The correlation between the two approaches was measured, considering the three types of alloreactivity (**Figure 5**). In the whole cohort, the phenotypically defined alloreactive NK cell subsets showed a strong and significant correlation with the percentage of lytic clones determined by cytotoxicity assays, albeit in most instances with higher values of the first over the second approach (**Figure 5A**, R=0.512, *p*<0.05). When we excluded LIR1^+^ cells from the phenotypically defined alloreactive NK cell subset, the correlation improved (**Figure 5B**, R=0.637, *p*<0.05). The strongest correlation was observed when the analysis was further restricted to Allo C2 and Allo Bw4 (**Figure 5C**, R=0.633, *p*<0.05), especially if LIR1^+^ cells were excluded (**Figure 5D**, R=0.741, *p*<0.05**)**. Specifically, when the analysis was restricted to Allo Bw4 only, a very good correlation of these parallel results was observed, primarily upon the corrective action of excluding LIR1^+^ cells from the alloreactive NK subset (**Table 2**). In Allo C2, there was again a good correlation with only one exception, represented by the D of patient #10. In this case, the frequency of lytic clones (18%) widely exceeded the size of the alloreactive subset (1%). A possible explanation could be the high representation of KIR2DL1^+^ KIR2DS2^+^ KIR2DL2/L3^−^ KIR3DL1^−^ NKG2A^−^ cells that cannot be quoted by phenotypic analysis at the population level (**Figure 3D**). Therefore, we analyzed by flow cytometry the phenotype of some of the lytic *vs*. non-lytic NK cell clones using various anti-KIR and anti-NKG2A mAb (**Figures 6A, B**). Lytic clones (i.e., A1-6) were KIR2DL1^+^ (143211^+^), some of which were co-expressing KIR2DS2 (1F12^+^/ECM41^−^). Due to their alloreactivity, we hypothesized the absence of KIR2DL2 in these clones. The evidence of NKG2A expression in 4 out of 6 lytic clones suggested an inefficient inhibition by this receptor upon the interaction with HLA-E on patient #10 PHA-blasts. It should be outlined that a weak HLA-E expression on PHA-blasts obtained from patient #10 was observed (**Supplementary Figure 4**). Moreover, the non-alloreactive NK cell clones (i.e., NA1-6) primarily expressed the iKIR recognizing patient KIR-L, as KIR3DL1 and/or KIR2DL2/L3.

In Allo C1, a weaker correlation was observed, and the size of the alloreactive subset was always greater than the frequency of the alloreactive NK cell clones (**Figure 5E**, R=0.304, *p*<0.2). This discrepancy could partially be corrected by the exclusion of LIR1^+^ cells (**Figure 5F**, R=0.326, *p*<0.1). We analyzed the phenotype of some clones derived from D1 and D2 of patient #11 (**Figure 6C**), showing expected results, as the unique expression of KIR2DL2/L3/S2 in lytic clones, while the presence of KIR2DL1/KIR3DL1 and/or NKG2A in non-lytic clones. The phenotype of D of patient #16 NK cell clones was also similar (**Figure 6D**). In this last set of clones, we also wanted to distinguish KIR2DL2/S2 from KIR2DL3, and we investigated the presence of LIR1. All three tested alloreactive NK clones expressed KIR2DL2 and not KIR2DL3, but this selectivity could be explained by the small number of phenotypically analyzed clones. The unexpected results were related to LIR1 expression observed both in lytic and non-lytic clones and the apparently identical phenotype of these alloreactive clones and the non-alloreactive NA2 clone.

The cytofluorimetric evaluation of the alloreactive NK subset correlates with the frequency of lytic clones. The receptor repertoire of lytic versus non-lytic NK clones mostly confirmed our knowledge of KIR or NKG2A recognition but also provided new insights.

## 4 Discussion

Adoptive immunotherapy based on alloreactive NK cells from haploidentical donors has proven efficacy and safety in the treatment of elderly AML patients with poor prognosis and not eligible for allogeneic HSCT. Importantly, the infused NK cells should include an appropriate amount of alloreactive NK cells, on the basis of the frequency of donor NK cell clones capable to kill patient-derived cells, to exert a crucial anti-leukemia effect. Thus, a “functional dose” of at least 2 x 10^5^ alloreactive NK cells/kg has been identified to predict clinical response (32, 33). More recently, this approach has also been extended to AML patients, eligible for allogeneic HSCT, who achieved CR after conventional chemotherapy but with persistent MRD positivity. At the current state of art, there was a need to identify a prompter but still reliable assay to measure the frequency of alloreactive NK cells, according to the model of KIR/KIR-L mismatch in donor-*vs*-recipient direction. Hence, the goal of this study was to perform parallel analyses of the NK cell repertoire of potential donors through i) NK cell cloning combined with cytotoxicity assays (i.e., the refer methodology in previous clinical studies) and ii) the multi-color cytofluorimetric analysis of freshly derived NK cells. Different pros and cons of the two approaches can be discussed. Referring to the first one, the possibility to detect the frequency of NK cells cytotoxic against patient-derived cells represents a valuable direct functional measurement. Another advantage of this method is that these results can be independent from the theory of all receptor/ligand interactions, which can still be incomplete and/or more complex than we routinely consider, as well as evaluating allelic polymorphism. However, this approach reveals some limitations that should be taken into account, mainly referring to the long time necessary to obtain the results (~6 weeks), and the requirement of strong expertise to perform high-efficiency NK cell cloning and ^51^Chromium-release assay. In addition, one should be sure that there is no preferential growth of certain cell types over the others. Another problem associated with long-term culture in the presence of cytokines can be an enhanced expression of CD94/NKG2A (43), which can inhibit the lysis of HLA-E^+^ target cells. The alternative method, which consists in the assessment of the phenotypically defined alloreactive NK cell subset, has the great advantage to be very rapid (1 day) and highly reproducible by many labs, especially if a commercially available kit becomes available. On the other hand, limitations concern the anti-KIR mAb specificity and the promiscuous ligand recognition of certain KIRs (see below). Moreover, there are still limited notions on the role of LIR1 inhibitory effect, as well as the possible intervention of other still unknown receptors. Nevertheless, when we associated the phenotypic analysis with functional degranulation assays evaluating the CD107a expression on shortly activated NK cells upon co-culture with patient-derived cells, we consistently demonstrated that the alloreactive subset was endowed with the highest activity. Despite the described caveats, it should be underlined that the results of the two approaches displayed a good correlation.

The various features related to the three types of alloreactivity will be discussed in more detail. In case of donor/recipient combination with HLA-C1 mismatch, each donor can always present the alloreactive subset (Allo C1) because the relevant iKIR(s) (KIR2DL2 and/or KIR2DL3) is/are always present. It should be reminded that KIR2DL2/L3 bind with low-affinity also HLA-C2, leading to a reduction of the alloreactive capability (19, 41). Indeed, we found in almost all cases that the size of the phenotypically defined alloreactive subset exceeded the frequency of lytic clones. We wondered whether LIR1, a weak inhibitory receptor, with broad HLA specificity, that can be expressed by a fraction of NK cells as well as by other lymphoid and myelomonocytic cells (42, 44, 45), could play a role, especially in Allo C1. Weak inhibitions delivered by both KIR2DL2/L3, upon low affinity recognition of HLA-C2, and LIR1 would add up, resulting in abrogation of lysis. Indeed, considering LIR1 as an additional inhibitory receptor, the alloreactive subset could be resized, improving the correlation between the two methodologies (**Table 2**). However, unexpected results were obtained by analyzing the receptor pattern of NK cell clones derived from D of patient #16 (**Figure 6D**). Indeed, both A1-3 and NA2 clones showed expression of KIR2DL2 and LIR-1. A possible explanation could be the differential expression of activating receptors responsible for the “ON” signal (e.g. NCRs) (46, 47) and/or the powerfulness of cytolytic machinery (48). Therefore, it can be envisaged that, in the presence of weak inhibitory interactions, only highly efficient NK cells will lyse the target cells. In addition, other relevant receptor(s) not considered in this study, might be differentially expressed in the A1-3 versus NA2 clones. Among the known HLA-specific receptors, the inhibitory KIR3DL2 could be excluded because neither HLA-A*03 nor –A*11 is present in this donor/patient pair (both have HLA-A*02 and –A*30), and KIR2DS4 was not expressed on the cell surface (only the deletion variant is present). The possible intervention of other still unknown receptors cannot be excluded.

When Allo C2 is expected by the donor/recipient HLA analysis, most donors will display an NK alloreactive subset because *KIR2DL1* gene is missing only in ~3-5% of the Caucasian population. KIR2DL1 has an exclusive specificity for HLA-C2, and a very good correlation between the two methodologies was observed. The only exception was represented by D of patient #10. In this case, the discrepancy in the frequency of alloreactive NK cells (1% by phenotype versus 18% by clonal analysis, **Table 2**) could be explained by the presence of KIR2DS2 leading to an under-estimation of the phenotypically defined alloreactive subset because KIR2DL1^+^ KIR2DS2^+^ NK cells cannot be quoted at the cell population level (19). Even though some new anti-KIR mAbs with mono-specific staining have been produced, as the recently described anti-KIR2DL1 specific HP-DM1 mAb (49, 50), an antibody recognizing KIR2DL2 but not KIR2DS2 is lacking, and *KIR2DL2* and *KIR2DS2* are in linkage disequilibrium (13). We tried to address this issue by analyzing the phenotype of D of patient #10 NK cell clones (**Figures 6A, B**). We proved that all six lytic clones were KIR2DL1^+^, four of which co-expressed KIR2DS2 without KIR2DL3 (1F12^+^/ECM-41^–^). The presence of KIR2DL2 would be phenotypically addressed only in KIR2DS2^−^/L3^−^, namely GL-183^+^ (or CHL^+^) and 1F12^−^ clones, and eventually hypothesized only on non-lytic clones. Indeed, KIR2DL2 strongly reacts with HLA-C1, efficiently inhibiting the lysis of HLA-C1^+^/C1^+^ patient’s PHA-blasts. Only a PCR analysis might be informative to assess the possible presence of KIR2DL2 in NK cell clones, but it was not performed in this present study. Another piece of information from the analysis at the clonal level regarded the relatively weak inhibition delivered by CD94/NKG2A as some lytic clones were NKG2A^+^. This observation was quite unexpected because the interaction between NKG2A and HLA-E should lead to NK cell inhibition toward all HLA class I^+^ cells. Indeed, HLA-E surface expression depends on binding nonameric peptides cleaved from the leader sequences of HLA-A, -B, -C. However, the strength of NKG2A/HLA-E inhibitory interactions can be variable, and a role of the dimorphism in *HLA-B* leader sequence at residue -21, encoding a strong binding methionine (-21M) and a weak binding threonine (-21T), has been described (51). This can determine different HLA-E expression and NKG2A^+^ NK cell education, higher in individuals carrying at least one -21M (-21M/x) than -21T/T *HLA-B* alleles (51, 52). In line with this knowledge, PHA-blasts derived from patient #10, characterized by -21T/T (HLA-B*58:01/49:01), showed a weak HLA-E expression compared to a -21M/M individual (i.e. patient #12, HLA-B*07:02/*14:02) (**Supplementary Figure 4**).

Differently from the other types of alloreactivity, Allo Bw4 can often be bypassed because of the relatively frequent absence of KIR3DL1 on the NK cell surface (26). This also occurred in one case of our cohort because, despite the Bw4 mismatch (**Table 1**), the available donor NK cells missed KIR3DL1 (i.e., D of patient #5, **Figure 1A**). Another limitation of this alloreactivity is the usual low frequencies of single-KIR3DL1^+^ cells, as observed in this cohort as well as in our past experience (23). This can be in line with the notion that NKG2C^+^ adaptive NK cells, expanded in response to CMV, also co-express KIR2DL specific for self-HLA-C and not KIR3DL1 (53). Finally, to correctly evaluate Allo Bw4, we believe that including HLA-*A23, *A24, and *A32 as KIR3DL1-ligands is mandatory, even though the KIR-ligand calculator website disregards them.

In addition to causing a lack of the receptor on the cell membrane (e.g., KIR3DL1*004, KIR2DS4 delta), *KIR* polymorphisms can influence KIR expression levels, the affinity with the ligands, and the strength of inhibitory signals, as described for KIR2DL1 (54), KIR2DL2/L3 (41, 55), and KIR3DL1 (56, 57). For an accurate study of NK alloreactivity the analysis of *KIR* genes at the highest resolution using next-generation sequencing (58), will become an important tool, when more widely available.

If KIR/HLA genetics are primarily relevant to understanding if a haploidentical donor can be NK alloreactive, the actual frequency of this subset in the total NK cell repertoire should be evaluated by phenotypic and/or functional assays. These studies allow the choice of the most suitable donor, including the identification of “superdonors” (59), whose NK repertoire reveals the highest frequency of alloreactive NK cells, and also the quantification of the “functional NK cell dose” to be infused into the patient. In conclusion, the systematic parallel study of NK alloreactivity and the critical view of the two approaches add new insights into the criteria for donor selection in the haploidentical setting. Indeed, the cytofluorimetric evaluation of the alloreactive NK subset well correlates with the frequency of lytic clones, with some advantages in terms of rapidity of the results and feasibility in multiple centers.

## Data availability statement

The raw data supporting the conclusions of this article will be made available by the authors, without undue reservation.

## Ethics statement

The studies involving human participants were reviewed and approved by CER-Liguria (Prot. n. 405REG2017) and CE-AVEC (Prot. n. 35/2017/O/Sper) (“NK-AML”); by CE-AVEC (Prot. n. 214/2018/Sper/AOUBo) (“MRD-NK”). The patients/participants provided their written informed consent to participate in this study.

## Author contributions

DP, RM, and LR designed the study, analyzed and interpreted the data. RM, PC, FL, and NC-C processed samples, performed phenotypic and functional analyses. SC processed samples, produced NK cell clones, evaluated their cytolytic activity, and performed KIR genotype. AB, SL, and AS performed leukapheresis manipulation and NK cell separation. CR provided 1F12 mAb. FG and PM performed correlation analyses and interpreted statistical results. FG, PM, LZ, and SP collected written consent of the patients and haploidentical donors and provided samples. AC and RML coordinated the clinical trials. DP and RM wrote the original draft of the manuscript. LR, FG, PM, AC, and RML contributed to manuscript preparation and critically revised the manuscript. RM and FG prepared the figures. DP, RM, PM, LR, and AC provided fundings. All authors contributed to the article and approved the submitted version.
