# Supplementary material for: Donor selection for adoptive immunotherapy with NK cells in AML patients: Comparison between analysis of lytic NK cell clones and phenotypical identification of alloreactive NK cell repertoire
Source: Front Immunol. 2023 Feb 14;14:1111419. doi: 10.3389/fimmu.2023.1111419 (PMC9971917; doi:10.3389/fimmu.2023.1111419)
Supplement: Supplementary file 1 [file Presentation_1.pdf]

SUPPLEMENTARY MATERIAL

1.1 Supplementary Figures

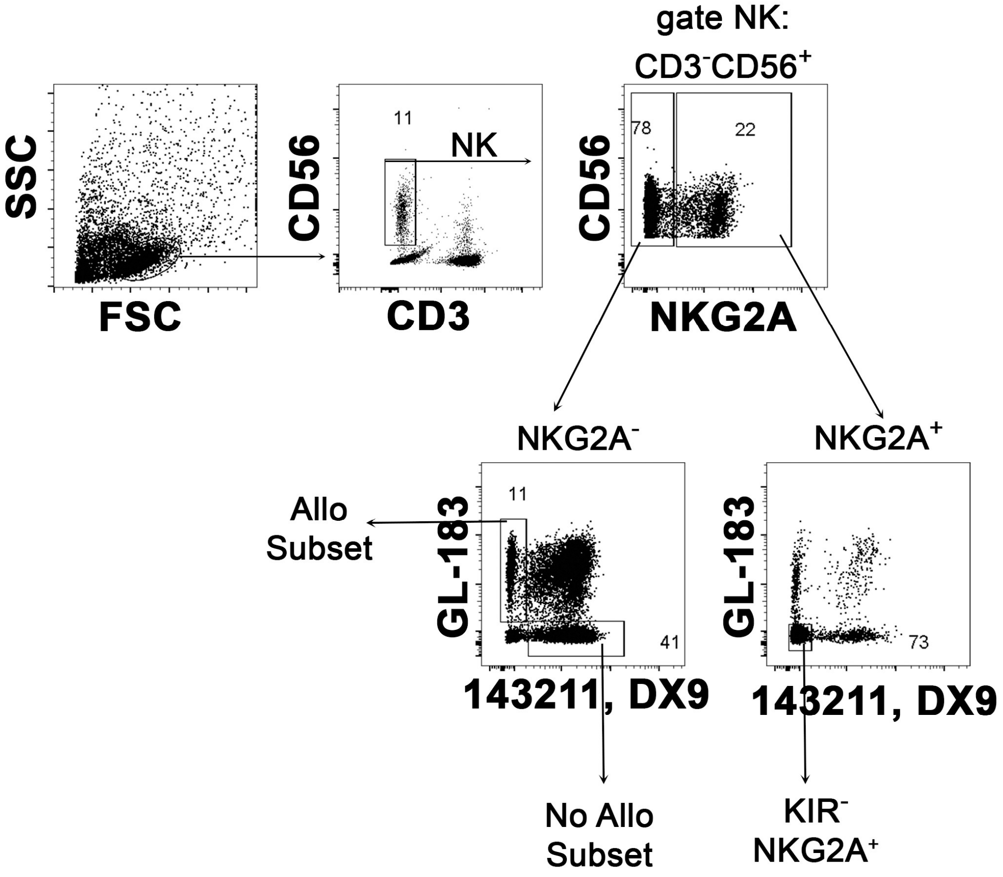

Supplementary Figure 1. Gating strategy to define NK cell subsets in degranulation assays.

The gating strategy to identify different NK cell subsets for degranulation assays of a representative donor (D of pt#16) is shown. $CD3^{-}$ $CD56^{+}$ NK cells can first be dissected into $NKG2A^{-}$ and $NKG2A^{+}$ cells. Among $NKG2A^{-}$ NK cells, appropriate anti-KIR mAb combinations allow to define the alloreactive subset (Allo Subset), the non-alloreactive subset (No Allo Subset). Among the $NKG2A^{+}$ NK cells, the indicated region identifies the $KIR^{-}NKG2A^{+}$ subset.

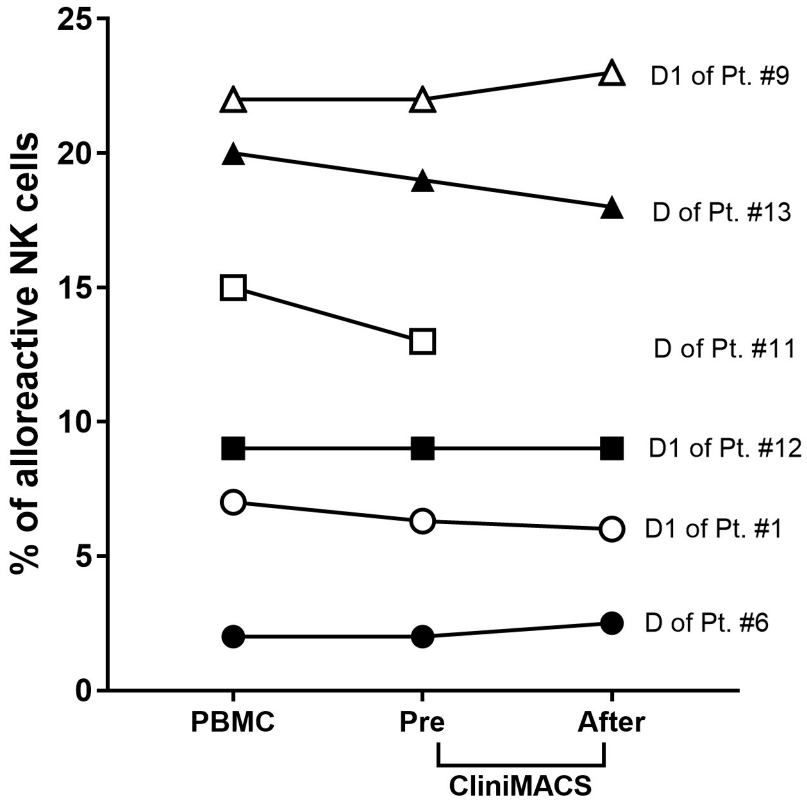

Supplementary Figure 2. Percentage of the alloreactive NK cell subset on PBMC (*n*=6), leukapheresis (pre-CliniMACS, *n*=6) and purified NK cells (after-CliniMACS, *n*=5), in different donors (of the group of patients infused at IRCCS Ospedale Policlinico San Martino, Genova). Gate: $CD3^{-}$ $CD56^{+}$ $CD45^{+}$ lymphocytes.

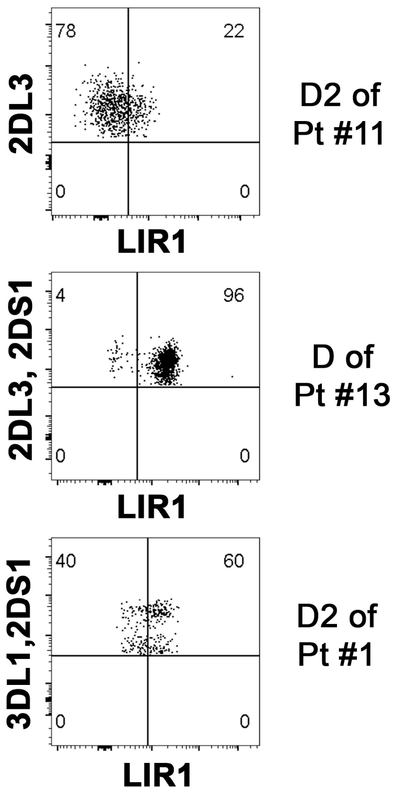

Supplementary Figure 3. Evaluation of the alloreactive NK cell subset excluding $LIR1^+$ cells. In three representative donors, the expression of LIR1 gating on the alloreactive cell subset is shown.

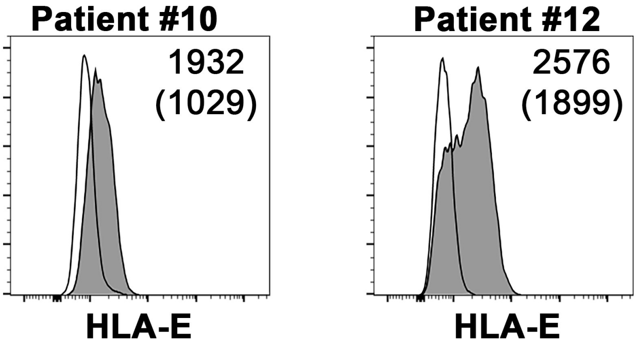

Supplementary Figure 4. Cytofluorimetric analysis of HLA-E expression on PHA-blasts derived from two patients. The staining was performed using 3D12 mAb followed by anti-mouse IgG1-PE (Southern Biotech) secondary reagent. Negative controls (i.e. cells only stained with the secondary reagent) are shown as empty histograms. Numbers and numbers in brackets represent the MFI and ΔMFI (i.e. the difference between MFI of cells stained with 3D12 and MFI of negative control), respectively.

1.2 Supplementary Tables

Supplementary Table 1: List of antibodies.

Clone	Specificity	Fluorochrome	Supplier/Reference
UCHT1	CD3	PE-CF594, BV510	BD Bioscience, San Josè, CA USA
BW264/56	CD3	VioBlue	Miltenyi Biotec, Bergisch Gladbach Germany
NCAM 16.2	CD56	BV421	BD Bioscience
N901	CD56	PE-Cy7	Beckman Coulter, Brea, CA USA
5B1	CD45	APC-Vio770	Miltenyi Biotec
CHL	KIR2DL2/S2/L3	FITC	BD Bioscience
H4A3	CD107a	PE	BD Bioscience
EB6B[§]	KIR2DL1/S1 and KIR2DL3*005	PE, PE-Cy7	Beckman Coulter
GL-183	KIR2DL2/S2/L3	PE, PE-Cy7, APC	Beckman Coulter
Z27	KIR3DL1/S1	PE, APC	Beckman Coulter
FES172	KIR2DS4	APC	Beckman Coulter
Z199	NKG2A	PE, APC	Beckman Coulter
REA110	NKG2A	FITC	Miltenyi Biotec
DX9	KIR3DL1	FITC, PE-Vio770	Miltenyi Biotec
143211	KIR2DL1, 2DS5	FITC, PE	R&D systems, Minneapolis, MN USA
HP-F1	LIR1 (CD85j)	APC	ThermoFisher, Waltham, MA USA,
ECM-41	KIR2DL3 (no *005)	Unconjugated (IgM)	(36, 37)
1F12	KIR2DS2 and KIR2DL3	Unconjugated (IgG2b)	(38)
3D12	HLA-E	Unconjugated (IgG1)	Biolegend, San Diego, CA USA

[§] EB6B has been indicated as EB6 in the text

Supplementary Table 2: Antibody combinations used to identify the alloreactive NK cell subsets.

Type of alloreactivity#	Permissive iKIR	Antibody combinations*		
		PE-conjugated	FITC-conjugated	APC-conjugated
Allo C1	KIR2DL2/L3	GL-183§	143211, DX9, NKG2A	LIR1¶
Allo C2	KIR2DL1	EB6B	CHL, DX9, NKG2A	LIR1¶
Allo Bw4	KIR3DL1	Z27§	143211, CHL, NKG2A	LIR1¶

# Defined on the basis of KIR-L mismatch (present in the donor and missing in the patient).
* Appropriate fluorochrome–conjugated anti-CD3 and anti-CD56 mAb combinations were also used to identify NK cells in PBMC.
§ In KIR2DS1$^+$ donors, EB6-PE can be also added in the alloreactive NK cell subset.
¶ Optional.
The size of the alloreactive NK cell subset is calculated as the percentage of PE-positive and FITC-negative cells.

Supplementary Table 3: Antibody combinations used in degranulation assay to identify different NK cell populations, including the alloreactive NK cell subset.

Type of alloreactivity	Permissive iKIR	Antibody combinations*		
		PC7-conjugated	FITC-conjugated	APC-conjugated
Allo C1	KIR2DL2/L3	GL-183§	143211, DX9	NKG2A
Allo C2	KIR2DL1	EB6B	CHL, DX9	NKG2A
Allo Bw4	KIR3DL1	Z27§	143211, CHL	NKG2A

§In KIR2DS1$^+$ donors and HLA-C2$^+$ patients pairs, EB6-PC7 was also added to include, in the alloreactive NK cell subset, this aKIR.
